# An FDA bioinformatics tool for microbial genomics research on molecular characterization of bacterial foodborne pathogens using microarrays

**DOI:** 10.1186/1471-2105-11-S6-S4

**Published:** 2010-10-07

**Authors:** Hong Fang, Joshua Xu, Don Ding, Scott A Jackson, Isha R Patel, Jonathan G Frye, Wen Zou, Rajesh Nayak, Steven Foley, James Chen, Zhenqiang Su, Yanbin Ye, Steve Turner, Steve Harris, Guangxu Zhou, Carl Cerniglia, Weida Tong

**Affiliations:** 1 Z-Tech Corporation, an ICF International Company at National Center for Toxicological Research, Food and Drug Administration, Jefferson, AR, USA; 2 Center for Food Safety and Applied Nutrition, Office of Applied Research and Safety Assessment, Division of Molecular Biology, Food and Drug Administration, Laurel, MD, USA; 3 Bacterial Epidemiology and Antimicrobial Resistance Research Unit, Richard B. Russell Research Center, Agriculture Research Service, U.S. Department of Agriculture, Athens, GA, USA; 4 National Center for Toxicological Research, Food and Drug Administration, Jefferson, AR, USA

## Abstract

**Background:**

Advances in microbial genomics and bioinformatics are offering greater insights into the emergence and spread of foodborne pathogens in outbreak scenarios. The Food and Drug Administration (FDA) has developed a genomics tool, ArrayTrack^TM^, which provides extensive functionalities to manage, analyze, and interpret genomic data for mammalian species. ArrayTrack^TM^ has been widely adopted by the research community and used for pharmacogenomics data review in the FDA’s Voluntary Genomics Data Submission program.

**Results:**

ArrayTrack^TM^ has been extended to manage and analyze genomics data from bacterial pathogens of human, animal, and food origin. It was populated with bioinformatics data from public databases such as NCBI, Swiss-Prot, KEGG Pathway, and Gene Ontology to facilitate pathogen detection and characterization. ArrayTrack^TM^’s data processing and visualization tools were enhanced with analysis capabilities designed specifically for microbial genomics including flag-based hierarchical clustering analysis (HCA), flag concordance heat maps, and mixed scatter plots. These specific functionalities were evaluated on data generated from a custom Affymetrix array (FDA-ECSG) previously developed within the FDA. The FDA-ECSG array represents 32 complete genomes of *Escherichia coli* and* Shigella.* The new functions were also used to analyze microarray data focusing on antimicrobial resistance genes from *Salmonella* isolates in a poultry production environment using a universal antimicrobial resistance microarray developed by the United States Department of Agriculture (USDA).

**Conclusion:**

The application of ArrayTrack^TM^ to different microarray platforms demonstrates its utility in microbial genomics research, and thus will improve the capabilities of the FDA to rapidly identify foodborne bacteria and their genetic traits (e.g., antimicrobial resistance, virulence, etc.) during outbreak investigations. ArrayTrack^TM^ is free to use and available to public, private, and academic researchers at http://www.fda.gov/ArrayTrack.

## Background

Foodborne pathogens are a leading cause of illness in the United States. In 1999, the Centers for Disease Control and Prevention estimated that there were around 76 million cases per year of illnesses due to foodborne agents, with 325,000 hospitalizations and 5,000 deaths in the United States each year [1]. More recent estimates suggest that the annual number of infections approaches 82 million [2].  Food contamination can occur at any step in the “farm-to-the-fork” continuum. Challenges in food safety stem from limited resources for surveillance of fresh and processed foods, sheer diversity, complexity of food matrices, and an increasingly globalized food supply chain. Recently, foodborne illness outbreaks have been increasingly recognized as a growing threat to the public health [3]. 

Food inspection and outbreak detection are two vital steps to ensure food safety. Since severe food-related outbreaks are usually caused by bacterial pathogens, it is crucial to have technologies that are capable of quickly detecting these pathogens with high sensitivity and reliability. The conventional methods, which are frequently used in food laboratories, are based on cultural, serological, and biochemical properties of specific bacterial pathogens. These phenotypic methods are time-consuming and labor-intensive. Moreover, they have limited utility for epidemiologic analysis of pathogen transmission during outbreak investigations because of their poor discriminatory power for closely related strains. Several genotyping methods have been developed and applied to provide estimates of genetic relatedness and make inferences about the outbreak transmission [4].

Typing methods such as pulsed-field gel electrophoresis (PFGE) that utilize restriction fragment analysis or polymerase chain reaction (PCR) are commonly used in epidemiological investigations of bacterial foodborne pathogens [4]. However, a major drawback of these typing methods is that they provide limited utility in understanding the genetic traits of bacterial strains, such as pathogenicity, virulence, or antimicrobial resistance. High-throughput microarray technology provides an effective way to identify, characterize, and obtain a nearly complete snapshot of the genetic repertoire of a particular isolate.  Such genome-wide insight is necessary for accurate and confident identification and discrimination of pathogens that may contaminate the food supply.

Microarray technology has been widely used in drug discovery and development, toxicology, and clinical application [5-9]. However, the use of this technology in detecting and characterizing foodborne pathogens is still in its infancy [10-18]. A properly designed genotyping microarray can not only provide strain-level discrimination within a particular pathotype, but also identify genetic elements responsible for virulence and antimicrobial resistance.  Furthermore, microarrays are highly parallel assays in that they can detect tens of thousands of genes simultaneously in a single experiment.  Combining this highly parallel assay with a semi-high-throughput workflow can provide a highly discriminatory and rapid subtyping method for use in epidemiological investigations of foodborne outbreaks. Indeed, the FDA has investigated several novel microarray-based strategies over the past several years in order to determine how best to identify and discriminate closely related strains [19]. In this manuscript, two separate microbial microarrays are examined, both of which were custom made by independent government agencies.  The FDA-ECSG array is a custom Affymetrix microarray developed by the FDA’s Center for Food Safety and Applied Nutrition and represents all of the genes found in 32 whole genome sequences and 46 related plasmid sequences from *Escherichia coli* and the related species, *Shigella *[20].  This array contains >23,000 independent genes and was designed to identify and discriminate between closely related strains of *E. coli* and *Shigella *[21] .  The second type of microarray examined in this study is a universal microarray developed by USDA scientists to detect antimicrobial resistance genes in bacterial pathogens [18,22-24]. 

ArrayTrack^TM^, a bioinformatics tool developed by the FDA, has been expanded to support microbial microarray data. ArrayTrack^TM^ has provided a rich set of functionality to manage, analyze, and interpret gene expression data from mammalian organisms [25,26] and has been widely adopted by the research community and used for review of pharmacogenomics data in the FDA’s Voluntary Genomics Data Submission program [27]. The new expansion of ArrayTrack^TM^ provides functionality to support microbial genomics research using microarrays. For example, ArrayTrack^TM^’s libraries have been populated with bioinformatics data from public domains related to bacterial pathogen species [28]. Data processing and visualization tools have been enhanced with customized options to facilitate analysis of microarray data generated from the custom microarrays developed at the FDA and USDA. Specifically, at the time of this writing, three new functions have been developed and are particularly effective for analysis of these microarray data: flag-based hierarchical clustering analysis (HCA), a flag concordance (FC) heat map, and flag indicators in the mixed scatter plot.

For each of the microarray experiments discussed in this manuscript, total genomic DNA was extracted from purified cultures of independent bacterial isolates and used as the target material for hybridization to individual microarrays.  We used the term “sample” to denote such a DNA extract; for a bacterial isolate without replicates of microarray hybridization, “sample” is synonymous with “isolate”. This manuscript illustrates the microbial genomics specific functionality in ArrayTrack^TM^ through the case studies based on these two microarrays.

## Implementation

The analysis and visualization tools presented below (except for the Microbial Library) utilize the gene presence and absence calls (which are called flags in ArrayTrack^TM^). While these tools share many common features with the tools used in gene expression analysis based on intensity values, they are particularly relevant and effective for identification and characterization of foodborne pathogens in the field of microbial genomics.

### Microbial Library

The Microbial Library is the newest addition to ArrayTrack^TM^’s collection of libraries. Currently, it holds 270,000 gene records from a total of 84 strains: 30 *Escherichia coli,* 39 *Salmonella enterica*, 10 *Shigella spp.*, and 5 *Vibrio spp*. As a starting point, the Microbial Library is focused on these four bacterial genera, which are common foodborne pathogens. ArrayTrack^TM^ also holds microbial pathway information for over 50 strains from Kyoto Encyclopedia of Genes and Genomes (KEGG) and gene ontology information for the *E. coli* K12 substrain MG1655. The gene data for the Microbial Library were downloaded from the National Center for Biotechnology Information (NCBI) website and stored on a local FDA database server. 

**Figure 1:**
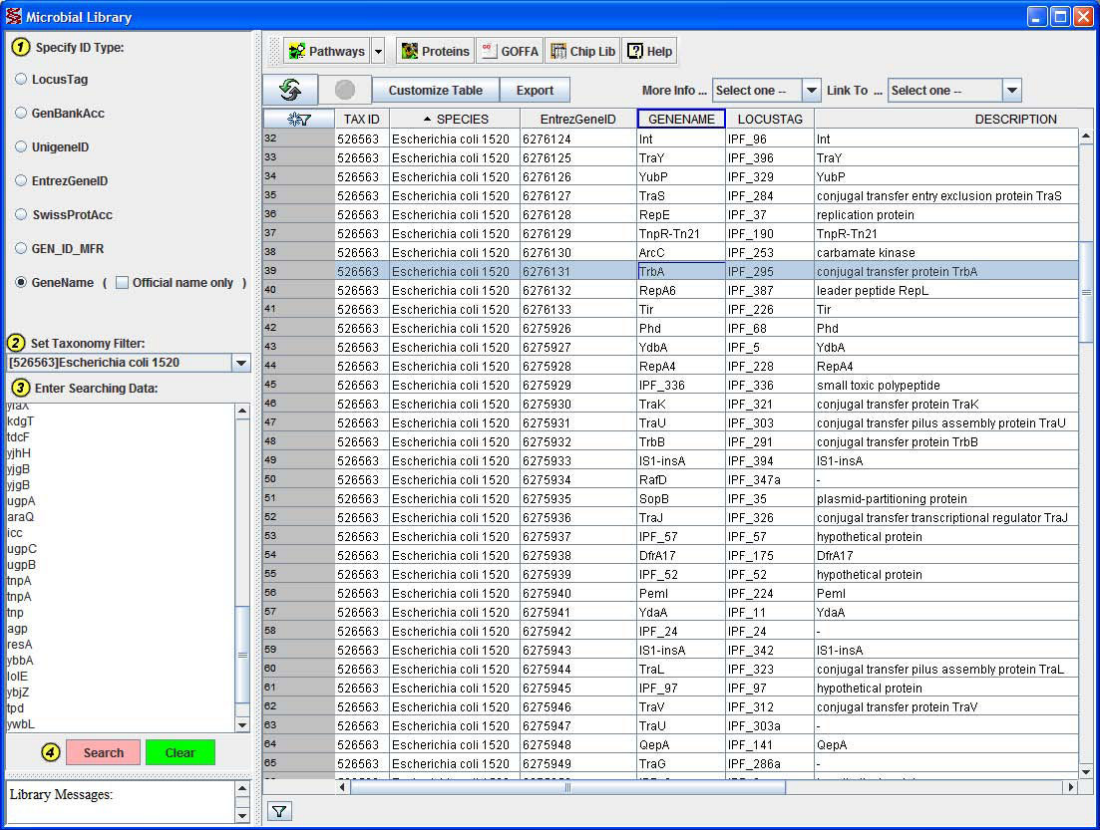
**Screenshot of ArrayTrack^TM^’s Microbial Library.** Among other functionalities, the center spreadsheet displays annotations for all the genes contained in the Microbial Library. The panel on the left can be used to simultaneously search for a specific list of genes.

As with other libraries in ArrayTrack^TM^, the Microbial Library provides an environment where scientists can search for a list of genes of interest by copying and pasting them to the Microbial Library’s search panel (on the left in Figure 1) rather than searching the NCBI website for one gene at a time.  As shown in Figure 1, the query results were displayed in a spreadsheet format which contains several columns of annotations for each gene including multiple identifiers (i.e., EntrezGeneID, GeneName, LocusTag), description, and gene type. The Microbial Library can be directly searched or filtered from ArrayTrack^TM^’s analysis functions, and its contents can also be highlighted and directly linked to other ArrayTrack^TM^ libraries and external websites (top panel in Figure 1). 

### Flag-based two-way Hierarchical Clustering Analysis (HCA)

**Figure 2:**
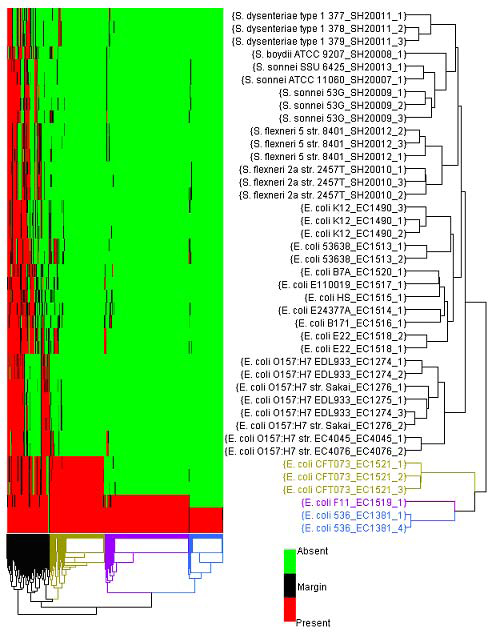
**Flag-based hierarchical clustering analysis of all 41 samples in the FDA-ECSG dataset.** Flag-based hierarchical clustering analysis of the FDA-ECSG data using a subset of 658 genes known to be associated with the *E. coli* 536 strain. The green and red colors within the HCA denote absent and present genes, respectively.  The bacterial isolates colored in blue, purple, and gold denote the *E. coli* 536_EC1381, *E. coli* F11_EC1519 and *E. coli* CFT073_EC1521 samples, respectively. Their strain-specific genes are also colored accordingly.

HCA is one of most widely used unsupervised analysis methods in pattern discovery. This tool has been an integral function in ArrayTrack^TM^ to conduct cluster analysis based on gene expression. In order to support the microbial genotyping data, the HCA has been expanded to operate on the similarity of flag values in different samples, whereas it previously only functioned with intensity values. Thus, the improved 2-way HCA is able to cluster similar strains together based on the presence/absence profile of genes, as well as cluster genes based on the similarity of different strains. An example of using this function in microbial genomics study is shown in Figure 2. 

The HCA function in ArrayTrack^TM^ offers extensive customization over the clustering process including multiple clustering algorithms, distance measurement options, visualization options such as coloring and scaling, filtering out non-informative genes with the same gene detection calls across arrays, re-clustering subsets, and clustering based on a subset of genes associated with specific characteristics of a particular strain.  A gene or group of genes can be selected and directly linked to ArrayTrack^TM^’s libraries to find more information about them. 

### Flag concordance (FC) heat map

**Figure 3:**
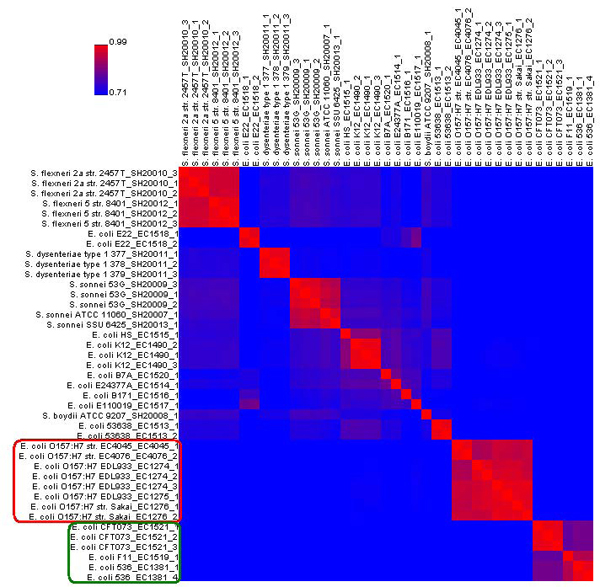
**Flag concordance heat map of all 41 *E. coli* and *Shigella* samples in the FDA-ECSG validation dataset** Heat map created using ArrayTrack^TM^’s default settings. The samples are reorganized using a clustering algorithm that places similar samples together. The areas in red within the heat map indicate high similarity. The *E. coli* O157:H7 samples are circled in red, while the *E. coli* 536_EC1381, *E. coli* F11_EC1519, and *E. coli* CFT073_EC1521 samples are circled in green. Both of these two groups are noted for their high within-group similarity values.

Measurement of similarity between two different bacterial strains is particularly helpful to microbiologists in indentifying outbreak strains and their relationships to known strains. The FC heat map is designed specifically to show the relatedness of strains and includes a visually insightful display of the results. The utility of this tool is illustrated in Figure 3 and explained in more detail below.

The FC heat map is a colored two-dimensional grid in which every sample is listed along both axes. Each cell is colored according to how similar the flag values of the two corresponding strains are to each other. The similarity value is based on the percentage of genes that share the same flag profiles, ranging from 0 to 100 percent. Colors are shades of purple, ranging from red (similar) to blue (different).  It should be noted that the data points on the main diagonal are always colored bright red (100 percent similarity) as it compares each sample to itself. The heat map is also symmetrical with respect to this diagonal.

The FC heat map can be used for quickly identifying which samples have similar absent/present calls to each other; ArrayTrack^TM^ automatically reorganizes the heat map using a clustering algorithm that places similar samples together. Groups of very similar samples will distinctly appear as blocks of red. The FC heat map can also be used by focusing on the row of a specific sample to identify which other samples are similar to it. 

There are several display options available for the FC heat map. The similarity threshold can be modified, changing how the grid is shaded. The size of the grid can also be adjusted. If desired, the clustering of the samples can be toggled off so that the samples are listed in the default order. Additionally, samples can be grouped together prior to the creation of the heat map, which will keep them adjacent to each other after clustering. 

### Mixed scatter plot

**Figure 4:**
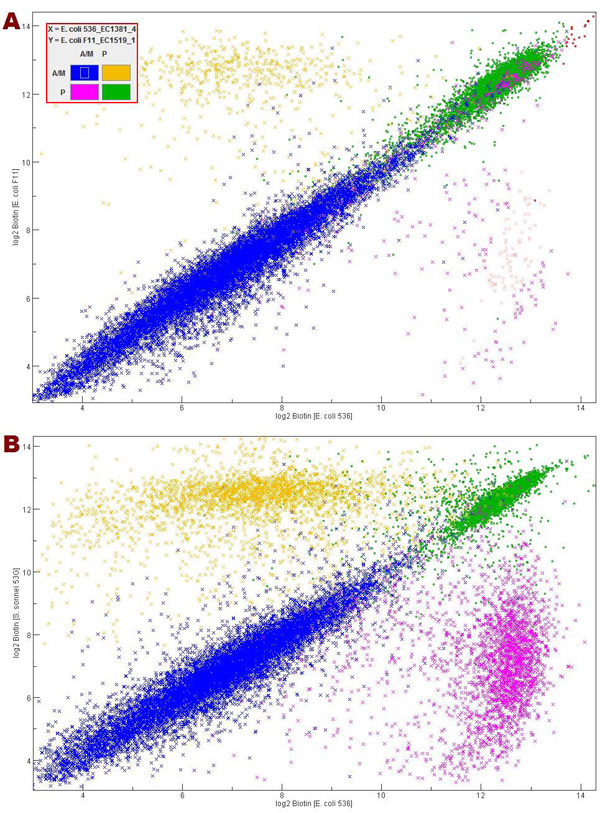
**Two mixed scatter plots from the FDA-ECSG dataset.** Mixed scatter plot **A** compares *E. coli* 536_EC1381 *vs.E. coli* F11_EC1519, and **B** compares *E. coli* 536_EC1381 *vs.Shigellasonnei* 53G_SH20009. Each gene was plotted on the graph based on its log_2_ intensity value in each sample, and color coded based on absent/marginal/present values according to the key in the upper left: pink genes are those considered to be present in the *E. coli* 536_EC1381 sample and absent/marginal in the other; yellow genes are absent in the *E. coli* 536_EC1381 sample and present in the other; blue genes are absent/marginal in both; and green genes are present in both.

The mixed scatter plot function compares two samples based on the intensity value, where the flag-based data is highlighted in different colors to differentiate genes that are absent in either one, both, or neither of the samples. An example of this tool is illustrated in Figure 4.

One of the most useful characteristics of the mixed scatter plot is its ability to highlight the genes that have significantly different probe intensities.  It can be used to identify genes with particularly high or low intensity values in either of the two samples as well as genes with intensity values that would not be expected from their flag value. The mixed scatter plot function in ArrayTrack^TM^ offers extensive functionality to assist the interpretation of the data displayed. For example, the display is customizable and supports changing the color scheme, range, scale, and flag-based filtering. One or more genes on the mixed scatter plot can be selected using the mouse and viewed in a spreadsheet or linked to various ArrayTrack^TM^ libraries. As with all of ArrayTrack^TM^ functions, the scatter plot can be exported as an image file.

## Results and discussion

ArrayTrack^TM^ has recently been expanded to incorporate tools that facilitate the analysis of microbial genomics data. In this manuscript, two microbial datasets were utilized as case studies to demonstrate how ArrayTrack^TM^’s new microbial genomics tools can effectively determine genetic differences between bacterial isolates. Both datasets are from independent government agencies and represent recent research efforts tailored towards food safety and food security as it applies to public health.

### Analysis of *E. coli* and *Shigella* strains using the FDA-ECSG microarray

The FDA-ECSG was designed to represent the broad global genomic diversity of *Escherichia coli* and the related species *Shigella spp*.  Included on this array are all of the genes present in 32 whole genomes and 46 plasmid sequences of these two species.  In total, approximately 23,000 unique gene targets were found from the whole genome sequences and each of these genes is represented on the FDA-ECSG as a probe set [21,29].  The FDA-ECSG dataset used in this case study was a validation set consisting of 41 hybridization experiments (CEL files) derived from 23 unique strains of *E. coli* or *Shigella* whose whole genome sequence had previously been determined.  By using sequenced reference strains as part of our validation study, we are able to assess the accuracy at which the platform calls genes present or absent.  Moreover, as part of the validation, biological replicates were performed on many of these strains to assess the reproducibility of the assay.   

After the raw CEL files were imported into ArrayTrack^TM^, gene presence/absence calls were made in ArrayTrack^TM^ using a modified version of the Affymetrix MAS5 gene detection algorithm.  The modifications to this algorithm were implemented after examining the validation data set and empirically determining the MAS5 parameters that provided the most accurate gene present/absent calls [20]. ArrayTrack^TM^’s FC heat map, flag-based HCA, and mixed scatter plot were used to analyze the data. 

The FC heat map of the FDA-ECSG dataset is shown in Figure 3. *E. coli* O157:H7 samples, circled on the left in red (Figure 3), exhibited a high flag-based similarity with each other but little similarity to other strains.  Thus the heat map demonstrated the extent of genomic diversity that exists within and between different *E. coli* pathotypes (O157:H7 *vs.* non-O157:H7). Additionally, it can be observed that the *E. coli* 536_EC1381, *E. coli* F11_EC1519, and *E. coli* CFT073_EC1521 samples (circled in green) were similar to each other, while distinct from other samples. Strains 536 and CFT073 belong to the same pathogen type (uropathogenic) and are distinct from the other strains examined.  

Statistical analysis tools in ArrayTrack^TM^ can be utilized on either all the genes or a selected subset of genes on a microarray. This flexibility is particularly useful for secondary, or “drill-down”, analysis in which the examination is first done on a global scale and then a fine-tuned investigation is carried out on a list of genes identified from the global analysis. For example, we performed flag-based HCA on the FDA-ECSG dataset using a list of 658 genes that are tailored to the *E. coli* 536 strain and included on the FDA-ECSG microarray. These genes were obtained by searching for Locus Tags containing ‘ECP’, which denotes the *E. coli* 536 strain, using the ArrayTrack^TM^’s Chip Library function. As depicted in Figure 2, the two samples from this strain are closely clustered together with genes (highlighted as blue) that are not present in other samples. This analysis identified which of the 23 strains are most similar to the *E. coli* 536 strain. For example, strains F11-EC1519 and CFT073-EC1521 (colored in purple and gold, respectively) are most similar to strain 536, which is consistent with the results of the FC heat map (Figure 3). 

Figure 4 shows two instances of FDA-ECSG data using the mixed scatter plot. As predicted by the FC heat map (Figure 3) and the HCA (Figure 2), there were fewer genes with differing flag values (pink or yellow spots) in Figure 4A comparing the two closely related *E. coli* strains than in Figure 4B comparing an *E. coli* strain to a *Shigella* strain. These scatter plots can be used to identify specific genes that exhibit differences between strains and link them to the Microbial Library (Figure 1) for annotations.

### Analysis of antimicrobial resistance genes in *Salmonella*

A series of universal microarrays have been developed by USDA scientists for detecting antimicrobial resistance genes in bacterial pathogens [18,22,23]. The latest version has 1,269 oligonucleotide probes to detect genes commonly associated with antimicrobial resistance and multi-drug resistant plasmids of the H1 and IncA/C lineages. An older version of the antimicrobial resistance chip that consisted of 775 resistance gene probes was used to study 34 *Salmonella* isolates from a turkey production facility [24]. Gene detection calls were made through an algorithm developed by USDA researchers who designed the chip [18,22] and the presence and absence calls were imported to ArrayTrack^TM^ for processing. Various functions in ArrayTrack^TM^ were used to identify the genes responsible for encoding the observed antimicrobial resistance in bacterial pathogens.

**Figure 5:**
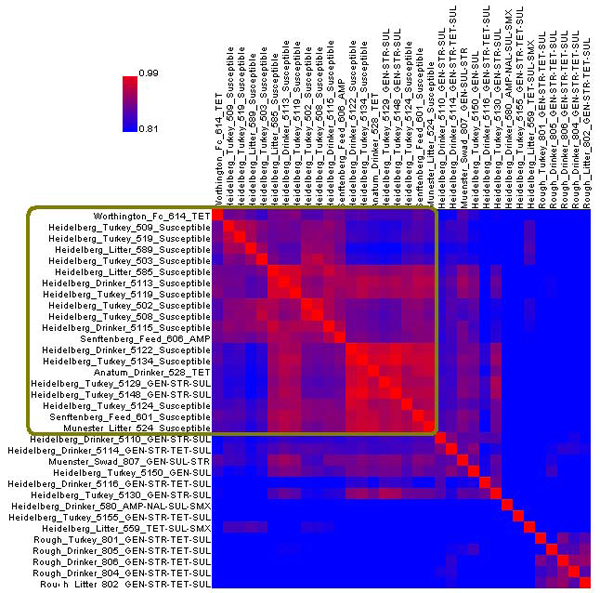
**Flag concordance heat map of 34 *Salmonella* isolates reorganized by ArrayTrack^TM^.** Red cells indicate a high percentage of matching gene absent/present calls while blue cells indicate a low percentage of matching gene calls. The gold outline indicates a subset of 20 isolates that show relatively high similarity to each other.

The FC heat map of the *Salmonella* isolates is shown in Figure 5. The heat map revealed distinct trends in the similarity of gene absent/present calls, focusing on the differences between isolates of *Salmonella* that are susceptible or resistant to antimicrobial drugs. In the upper left corner of the FC heat map, the large red block (circled in gold in Figure 5) represents a collection of 20 isolates that showed strong flag-based similarity; all 15 isolates that were susceptible to all antimicrobials tested were included in this block. The heat map analysis grouped the susceptible *Salmonella* isolates together and illustrated that the majority of antimicrobial resistant isolates did not group with the sensitive isolates. This suggests that the turkey facility in this study was populated by one group of mostly antimicrobial susceptible isolates and a diverse group of resistant isolates. The heat map analysis can be a useful tool for researchers to visualize the differences among the patterns of drug resistance in bacterial pathogens. 

**Figure 6:**
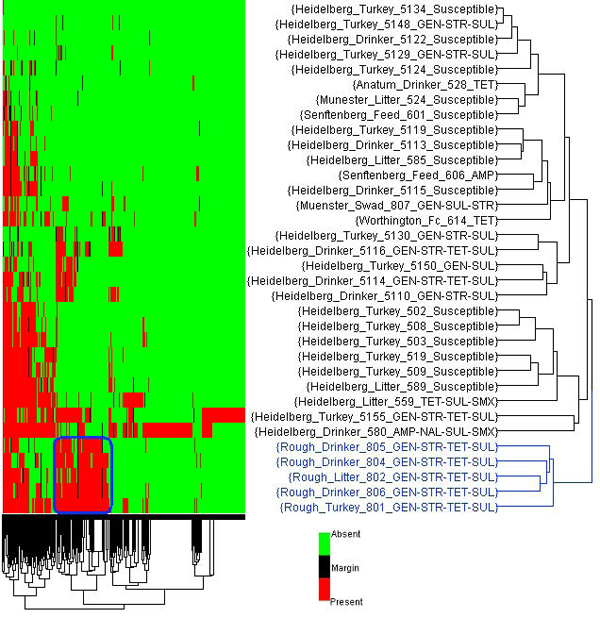
**Hierarchical clustering analysis of 34 *Salmonella* isolates**. Flag-based HCA of the 34 *Salmonella* isolates.  To identify the dissimilarity among isolates, only those genes (297) that showed differences in absent and present calls between the isolates were used.   The green and red colors indicate absent or present calls, respectively.  The isolates with a “Rough” serotype were colored in blue and their specific gene cluster is outlined in blue.

Figure 6 displays a flag-based two-way HCA based on gene detection calls performed on the 34 *Salmonella* isolates with all 297 genes that varied between isolates included. The HCA showed that the five *Salmonella* isolates with a “Rough” serotype were clearly separated from the other isolates. Their specific antimicrobial resistance genes were identified for further analysis, some of which could include the use of ArrayTrack^TM^’s additional tools and libraries. 

In summary, both cases studies demonstrate how the ArrayTrack^TM^ microbial genomics functions can be utilized to discover meaningful information in the microarray genotyping data for bacterial pathogens. The FC heat map, flag-based HCA, and mixed scatter plot tools were used to assist investigators in observing significant relationships among the isolates in terms of their genetic compositions, drug resistance profiles, and phenotypic information (e.g., demographics, sources, serotypes, etc.).  

## Conclusions

The ArrayTrack^TM^ platform has been extended to support microbial data with the addition of the Microbial Library and three functional tools (the flag concordance heat map, flag-based hierarchical clustering analysis, and mixed scatter plot). The software can be used as a one-stop solution for analyses enabling detection and characterization of bacterial foodborne pathogens. ArrayTrack^TM^ provides vital support for the FDA’s and other government agencies’ food protection efforts by augmenting investigators’ capabilities to rapidly identify bacteria and their genetic traits (e.g, antimicrobial resistance, virulence, etc.) during outbreak investigations. Lastly, ArrayTrack^TM^ is freely available on the web and its enhanced capabilities can be used by researchers to assess the threat of bacterial pathogens in accidental or deliberate outbreak scenarios.

## List of abbreviations used

ECSG: *Escherichia coli* and* Shigella*; FC: Flag Concordance; FDA: Food and Drug Administration; KEGG: Kyoto Encyclopedia of Genes and Genomes; HCA: Hierarchical Clustering Analysis; NCBI: National Center for Biotechnology Information; USDA: United States Department of Agriculture

## Authors’ contributions

HF coordinated the project, performed data analysis, and helped significantly to draft the manuscript. JX helped significantly to coordinate the project and draft the manuscript. DD created the first draft of the manuscript and performed data analysis. SJ and IP provided the FDA-ECSG data. JF, WZ, RN, and CC provided the antimicrobial resistance data. RN, SF, and WT helped draft the manuscript. SJ, IP, WZ, JC, JF, and CC revised the manuscript. ZS, YY, ST, SH, and GZ performed the software and database programming for ArrayTrack^TM^. WT helped coordinate the project and finalized the manuscript. All authors read and approved the final manuscript.

## Competing interests

The authors declare that they have no competing interests.
